# Skeletal and cardiovascular consequences of a positive calcium balance during hemodialysis

**DOI:** 10.1590/2175-8239-JBN-2020-0098

**Published:** 2020-10-26

**Authors:** Rosilene M. Elias, Sharon Moe, Rosa M. A Moysés

**Affiliations:** 1 Universidade de São Paulo, Hospital das Clínicas, Departamento de Medicina, Divisão de Nefrologia, São Paulo, SP, Brasil.; 2 Universidade Nove de Julho, São Paulo, SP, Brasil.; 3 Indiana University School of Medicine, Department of Medicine, Division of Nephrology, Indianapolis, Indiana, USA.; 4 Roudebush Veterans Administration Medical Center, Indianapolis, Indiana, USA.

**Keywords:** Chronic Kidney Disease-Mineral and Bone Disorder, Parathyroid, Calcium Transfer Mass, Vessels, Myocardium, Distúrbio Mineral e Ósseo na Doença Renal Crônica, Paratormônio, Massa de Transferência deCálcio, Vasos, Miocárdio

## Abstract

Patients on hemodialysis are exposed to calcium via the dialysate at least three times a week. Changes in serum calcium vary according to calcium mass transfer during dialysis, which is dependent on the gradient between serum and dialysate calcium concentration (d[Ca]) and the skeleton turnover status that alters the ability of bone to incorporate calcium. Although underappreciated, the d[Ca] can potentially cause positive calcium balance that leads to systemic organ damage, including associations with mortality, myocardial dysfunction, hemodynamic tolerability, vascular calcification, and arrhythmias. The pathophysiology of these adverse effects includes serum calcium changes, parathyroid hormone suppression, and vascular calcification through indirect and direct effects. Some organs are more susceptible to alterations in calcium homeostasis. In this review, we discuss the existing data and potential mechanisms linking the d[Ca] to calcium balance with consequent dysfunction of the skeleton, myocardium, and arteries.

## Resumo

Pacientes em hemodiálise são expostos ao cálcio, por meio do dialisato, pelo menos três vezes por semana. As alterações no cálcio sérico variam de acordo com a transferência de massa de cálcio durante a diálise, que é dependente do gradiente entre a concentração de cálcio no plasma e no dialisato (d [Ca]) e o estado de renovação do esqueleto que altera a capacidade do osso de incorporar cálcio. Embora subestimado, o d [Ca] pode potencialmente causar balanço positivo de cálcio que leva a danos em órgãos sistêmicos, incluindo associações com mortalidade, disfunção miocárdica, tolerabilidade hemodinâmica, calcificação vascular e arritmias. A fisiopatologia desses efeitos adversos inclui alterações do cálcio sérico, supressão do hormônio da paratireóide e calcificação vascular por meio de efeitos diretos e indiretos. Alguns órgãos são mais suscetíveis a alterações na homeostase do cálcio. Nesta revisão, discutimos os dados existentes e os mecanismos potenciais que ligam o d [Ca] ao equilíbrio do cálcio com a consequente disfunção no esqueleto, miocárdio e artérias.

## Introduction

Patients with end-stage kidney disease (ESKD) have increased morbidity and mortality^1^; chronic kidney disease mineral bone disorder (CKD-MBD) is a consistent independent risk factor. Although calcium disturbances and treatments that alter serum calcium have been addressed in several reviews, the impact of a positive calcium balance during hemodialysis due to calcium dialysate content - d[Ca] - on this outcome is rarely considered.

Calcium is an essential mineral for the function of various organ systems through its impact on hormones and cell signaling. As a result, humans have a complex homeostatic system to maintain normal levels of calcium in the blood through regulation by multiple hormones acting on the intestine, parathyroid, kidneys, and bones. With evolution from fish to amphibians, the skeleton had to adapt because, compared to the ocean, the calcium content in land food and water is lower. Therefore, the skeleton became an organ that not only gave motility and strength to the body, but also served as a reservoir of calcium that can respond, even at its own expense, to maintain normal levels of ionized calcium. Calcium accounts for 1 to 2% of adult human weight, and over 99% is found in teeth and bones. The ratio of extracellular to intracellular calcium and the amount of calcium stored within the cells are tightly controlled^2^.

**Figure 1:**
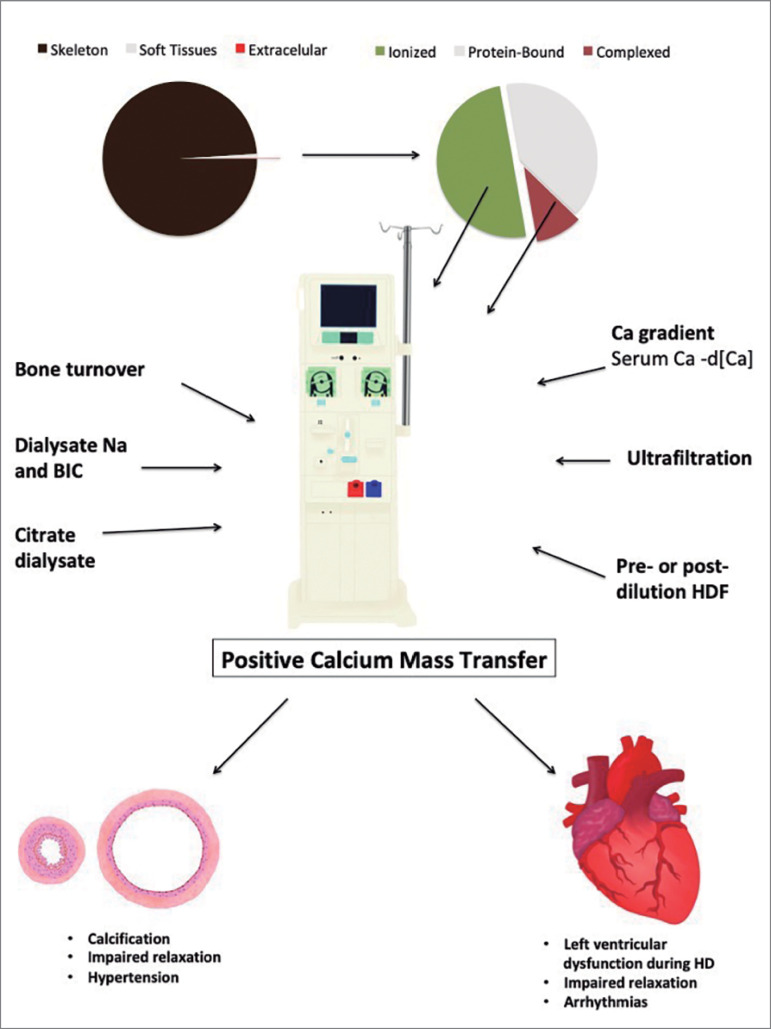
Simplified scheme of the several factors that influence calcium balance during hemodialysis and systemic consequences of d[Ca] on several organs. Mostly the calcium gradient between serum calcium and d[Ca], but also ultrafiltration and bone turnover status influence calcium mass transfer during hemodialysis. Calcium mass transfer and/or serum calcium determines systemic response in several sites, such as myocardial and vessels.

This homeostasis is disrupted in patients with chronic kidney disease (CKD) due to disruption of normal homeostatic loops with kidney function decline, with compensatory changes in several hormones (parathyroid hormone - PTH, vitamin D, fibroblast growth factor 23 - FGF23). The abnormalities of CKD-MBD begin in some patients as early as eGFR of 70 mL/min/1.73m^2^ and are almost universal when eGFR is < 30mL/min/1.73m^2^ 3. In the early 1970s, it was discovered that 1,25vitamin D levels were uniformly reduced, and calcium intestinal absorption was decreased in patients with CKD. The conclusion at that time was that patients with CKD were therefore in a negative calcium balance and thus would require calcitriol and/or calcium supplementation to enhance intestinal absorption and avoid hypocalcemia and secondary hyperparathyroidism. However, subsequent studies in patients with moderate CKD have shown that oral calcium carbonate supplementation^4^ or calcium-rich diet^5^ induce a positive overall calcium balance that might facilitate soft-tissue deposition of calcium salts. Furthermore, the discovery in the early 2000s that FGF23 inhibits calcitriol synthesis supports an attempt of the body to purposefully decrease intestinal calcium absorption, due to the marked reduction of calcium excretion with declining kidney function. These studies have led to the current concept that the decreased intestinal absorption is an appropriate adaptation to avoid positive calcium balance and its adverse consequences^6^. The need for hemodialysis adds a new variable to this broken regulatory system as patients are exposed to an external calcium-rich solution (dialysate) during each dialysis treatment. The acute change in ionized calcium during dialysis can alter the balance between bone and serum calcium, further altering overall calcium balance (Figure 1). The purpose of this review is to examine the evidence supporting a pathologic role of a positive calcium balance on end organ damage in patients undergoing hemodialysis.

### Calcium transport during hemodialysis

Calcium balance during dialysis is defined as the net amount of calcium that is obtained (or lost) during a treatment. Briefly, this balance is determined by two factors: the calcium gradient and the ultrafiltration volume. Calcium gradient is defined by the difference between dialysate and serum calcium. The blood calcium is composed of three fractions: protein bound, complexed, and ionized^7^. The ionized calcium is the active fraction representing ~50% of the total calcium. Only the ionized calcium and calcium complexed to small anions are transported across the dialyzer membrane, and the latter is only estimated and not usually measured. Albumin is the primary calcium-binding protein, but unfortunately, formulas estimating ionized calcium based on total calcium and albumin are inaccurate, particularly in dialysis patients^8^. Calcium can also bind to phosphate and bicarbonate, and concentrations of both can change markedly during dialysis. Therefore, the difference between dialysate and the blood-free calcium times the total dialysate volume is the main component of the calcium mass balance during the dialysis and could be either negative or positive. Conversely, there will be always a negative balance of calcium due to the ultrafiltration volume. In other words, the patients will always loose a small amount of calcium when we prescribe a negative balance of water. However, this is a small component of the calcium balance during dialysis, and the calcium gradient predominates.

Usually, the concentration of total calcium in the extracellular compartment increases during dialysis and decreases between the sessions. In most studies, the use of d[Ca] of 1.5 or 1.75 mmol/L (3.0 or 3.5 mEq/L) leads to post-dialysis hypercalcemia and positive calcium balance. However, the actual calcium mass transfer is unpredictable due to 1) differences in the ionized calcium blood level, 2) differences in convective loss or calcium that varies depending on ultrafiltration, and 3) imprecise calculations of the delivered dialysate calcium^9^^,^ 10. Finally, our work has shown that the change in ionized calcium is greatest in the first 30 to 60 minutes and also depends on bone remodeling^9^. Thus, not all patients will gain or lose calcium, or develop hyper or hypocalcemia on the same d[Ca] concentration. However, as shown in Table 1, many studies based their conclusion on intradialytic calcium balance using only the serum calcium variation. Moreover, some studies have shown that changing sodium concentration in the dialysate will decrease the calcium concentration affecting the intradialytic calcium mass transfer^11^.

**Table 1:** Summary of several studies showing calcium mass transfer (CMT) according to dialysate calcium concentration - d[Ca]

Author	Year	Method of dialysis/population	Buffer/ Dialysate	Measurements	Calcium Mass Transfer (CMT)
Ogden^52^	1966	HD with tank and coil; 5h; N =? / 25 sessions	Acetate/coil dialyzer	Estimated using differences between initial and final serum tCa	-d[Ca] of 1.125 mmol/L = -124 mg -d[Ca] of 1.375 mmol/L = 0 mg -d[Ca] of 1.75mmol/L = + 426 mg
Wing^53^	1968	HD with tank and Kiil; 12h; N = 1, 10 sessions	Acetate/Kiil dialyzer	Measurement of tCa in the total volume of dialysate	-Several d[Ca]s, from 0.738 to 1.988 mmol/L -CMT from -1,114 to + 740 mg
Goldsmith^54^	1971	HD with tank and Kiil; 6h; N = 5	Acetate	Measurement of tCa and 45Ca in samples of dialysate	-Fórmula proposta -Proposed Formula - Net gain [mg/min] = 0.108 + 0.623 x calcium gradient [mg/ml])
Strong^55^	1971	HD with tank and Kiil; 3-4h; N = 13	Acetate	Measurement of tCa, 47Ca and 45Ca in samples of dialysate	-d[Ca] of 1.475 mmol/L = 0 mg -d[Ca] of 1.725 mmol/L = +91 mg -d[Ca] of 1.975mmol/L = + 240 mg
Skrabal^56^	1974	HD with tank and coil; 8h; N = 3	Acetate	Measurement of tCa in samples of dialysate	-d[Ca] of 1.5 mmol/L = +72 mg -d[Ca] of 2 mmol/L = +240 mg
Carney^57^	1985	HD	Bicarbonate	?	-d[Ca] of 1.65 mmol/L = from -66 to +72 mg
Hou^58^	1991	HD, 4h; N = 7; Baxter SPS 550	Bicarbonate	Measurement of tCa in samples of dialysate	-d[Ca] of 0.75 mmol/L = -231 mg -d[Ca] of 1.25 mmol/L = 0 mg -d[Ca] of 1.75mmol/L = + 879 mg
Argilés^12^	1993	Post-dilutional HDF; 3h; N =9	Bicarbonate	Measurement of iCa in samples of dialysate.	-No calculation of CMT. Assumption that CMT is neutral with d[Ca] of 1.25 mmol/L, slightly + with 1.5 mmol/L, and significantly + with 1.75 mmol/L
Malberti^59^	1994	Post-dilutional HDF, 4h; N = 7	Bicarbonate	Measurement of tCa in the total volume of dialysate	-d[Ca] of 1.25 mmol/L = -44.8 mg for infusion rate = 2.5l/h and -56.8 mg for infusion rate = 5l/h -d[Ca] of 1.5 mmol/L = -23.6 mg for infusion rate = 2.5l/h and -22.8 mg for infusion rate = 5l/h -d[Ca] of 1.75mmol/L = -11.2 mg for infusion rate = 2.5l/h and -13.2 mg for infusion rate = 5l/h
Argilés^60^	1995	Post-dilutional HDF, 4 h; N = 14, proportion machine	Bicarbonate	Measurement of iCa and tCa in samples of dialysate	Using iCa: -d[Ca] of 1.25 mmol/L = neutral; d[Ca] of 1.5mmol/L = positive Using tCa: -d[Ca] of 1.25 mmol/L = negative; d[Ca] of 1.5mmol/L = neutral
Fabrizi^61^	1996	HD, 4 h; N = 6, proportion machine	Bicarbonate	Measurement of iCa in samples of dialysate	-d[Ca] of 1.25 mmol/L = -6 mg -d[Ca] of 1.75mmol/L = + 308 mg
Ding^62^	2002	Pre-dilution HDF, post-dilution HDF and acetate free HD/N=12	Bicarbonate/Citrate	Measurement of blood iCa and tCa	-CMT not measured -use of different d[Ca] for HDF and acetate free HD
Al-Hejaili^63^	2003	HD, 2, 4 and 6h; N = 14	Bicarbonate	Measurement of tCa in the total volume of dialysate	-d[Ca] of 1.25 mmol/L = -25 mg in 2h; -0.6 mg in 4 h and – 82 mg in 6h -d[Ca] of 1.75mmol/L = + 43 mg in 2h; 96 in 6h
Sigrist^64^	2006	HD; 4h; N = 52	Bicarbonate	Measurement of tCa in proportional samples of dialysate	-d[Ca] of 1.25 mmol/L = -187 mg (range: - 486 - + 784 mg)
Karohl^10^	2010	HD, 4h, N = 23, Genius Hemodialysis system	Bicarbonate	Measurement of tCa in a proportional sample of dialysate	-d[Ca] of 1.0 mmol/L = -492 mg -d[Ca] of 1.25 mmol/L = -468 mg -d[Ca] of 1.5 mmol/L = -46 mg -d[Ca] of 1.75mmol/L = + 268 mg
Basile^65^	2011	HD, 4 and 8h; N = 11; Genius Hemodialysis system	Bicarbonate	Measurement of iCa in proportional samples of dialysate	-d[Ca] of 1.5 mmol/L; 4h = + 285 mg; 8h = + 298 mg
Movili E	2011	HD switched to HDF (N=30 vs 35 control)	Bicarbonate	Effect of 6 months of HDF on serum Ca, P and PTH	-CMT not measured -Significant reduction of P and PTH concentrations with no significant changes in Ca
Bosticardo^66^	2012	HD; 4h; N = 22	Bicarbonate	Measurement of iCa in samples of blood and dialysate	-CMT not measured, but inferred -d[Ca] of 1.25 mmol/L = -57 mg (-288 to +110 mg) -d[Ca] of 1.5 mmol/L = +93 mg (-108 to +337 mg)
Basile^67^	2012	HD, 3-4h, N = 23, Genius Hemodialysis system	Bicarbonate	Measurement of iCa and tCa in a proportional sample of dialysate	Using iCa: -d[Ca] of 1.25 mmol/L = +97 ± 128 mg -d[Ca] of 1.375 mmol/L = +187 ± 146 mg -d[Ca] of 1.5mmol/L = +326 ± 253 mg Using tCa: -d[Ca] of 1.25 mmol/L = +75 ± 122 mg -d[Ca] of 1.375 mmol/L = +182 ± 125 mg -d[Ca] of 1.5mmol/L = +293 ± 228 mg
Grundstrom^14^	2013	HD N=9/HDF N=11	Bicarbonate/Citrate	Measurement of iCa in samples of blood	-CMT not measured -Citrate dialysis fluid resulted in lower post-dialysis plasma iCa (1.10 mM vs. 1.27 mM)
Safranek^68^	2015	HD 4h, N=80 and Post-dilutional HDF N=46	Bicarbonate/Citrate	Measurement of iCa and tCa in samples of blood	-CMT not measured -d[Ca] = 1.5 mmol/L: increases in iCa and tCa, less significant in acetate-free HDF
Bacchetta^11^	2015	HDF; 4h; N=28 children	Bicarbonate	Measurement of iCa and tCa in samples of blood	-CMT not measured -Decrease of tCa and iCa with D[Ca] 1.25 and no change with D[Ca] 1.5mmol/L - Increasing bicarbonate and/or decreasing Na requested in the dialysate decreases calcium extraction from the acid preparation
Tiranathanagul^69^	2015	HDF; 4h; N=22	Bicarbonate/Citrate	Measurement of iCa in samples of blood	-d[Ca] = 1.5 mmol/L: increase in iCa, more significant in acetate-free HDF
Waniewski^70^	2016	HD; 4h; N = 25	Bicarbonate	Measurement of tCa in proportional samples of dialysate	-d[Ca] of 1.35 mmol/L = -22.7 ± 54 mg
Goldenstein^9^	2018	HD; 4h; N=10	Bicarbonate	Measurement of tCa in proportional samples of dialysate	Pre PTX: -d[Ca] of 1.25 mmol/L = -89 mg (-180 to +23 mg) -d[Ca] of 1.5 mmol/L = -106 mg (-389 to +75 mg) -d[Ca] of 1.75 mmol/L = 12.8 mg (-7 to +169 mg) Late post-PTX: -d[Ca] of 1.25 mmol/L = -3 mg (-22 to +112 mg) -d[Ca] of 1.5 mmol/L = -29.7 mg (-103 to +248 mg) -d[Ca] of 1.75 mmol/L = 38 mg (-158 to +139 mg)
Di Filippo^18^	2018	HD; 3-5h; N = 34	Bicarbonate	Measurement of iCa in samples of blood and one sample of dialysate	-CMT not measured -d[Ca] of 1.25 mmol/L, assumption of a negative CMT based on blood iCa variation
Havlin^71^	2019	Post dilution HDF; 4h; N=10	Bicarbonate	Measurement of iCa in proportional samples of dialysate	- d[Ca] of 1.25 mmol/L, bic. 26 mmol/L= -309 mg - d[Ca] of 1.25 mmol/L, bic. 32 mmol/l=-108 mg

HD: hemodialysis; tCa: total calcium; P: phosphorus; PTH: parathyroid hormone; HDF: hemodiafiltration; iCa: ionized calcium; PTX: parathyroidectomy. For CMT, values are presented as shown in the original publication.

Interestingly, the majority of the studies on intradialytic calcium balance was done on hemodialysis (Table 1). However, hemodiafiltration has been adopted by several centers, sometimes as the main modality of renal replacement therapy. In this case, water and fluid change through convection might reach 30 liters and certainly gains importance in comparison with the maximum 3-4 liters that are extracted in hemodialysis. Conversely, the diffusion process has a lower impact on the calcium balance when compared to hemodialysis. Studies on post-dilutional hemodiafiltration done in the 1990s show similar results on calcium balance to those on hemodialysis^12^. However, the use of pre-dilutional hemodiafiltration can be associated with a negative intradialytic calcium balance. Pieces of evidence of long-term effects of hemodiafiltration, however, are scarce in the literature. Argiles et al.^12^ in 1993 compared the effects of lowering d[Ca] to 1.25 mmol/L in 7 patients vs. 6 control patients using d[Ca] 1.5 mmol/L. Calcium carbonate oral intake was more than doubled in the low d[Ca] group. Total calcium, phosphate, and alkaline phosphatase were similar in both groups over the year. Basile et al.^13^ showed in 2001 that neither pre- nor post-dialysis systolic and diastolic blood pressures, pre-dialysis serum bicarbonate and pH, and pre-dialysis serum sodium, potassium, calcium, or phosphorus were significantly different when comparing hemodialysis and hemodiafiltration.

Another potential confounding factor for the intradialytic calcium balance is the use of citrate instead of acetate in the dialysate. It has been demonstrated that PTH decreases and ionized calcium increases using bicarbonate buffer, whereas acetate does the opposite, increasing PTH and decreasing serum calcium^14^.

Despite the intradialytic effect on calcium balance, it is important to recognize the potential acute effect of dialysis on serum calcium. It is plausible that acute changes in extracellular calcium may affect intracellular calcium concentration, which is a key signaling pathway for nearly every cell. In animal models of CKD, there is an increase in intracellular calcium concentration in multiple cell types. In cardiomyocytes, the increase is mediated by PTH through a process that includes both increased entry of calcium into cardiac myocytes and decreased exit of this ion from these cells^15^. There is also an increase of basal intracellular calcium in vascular smooth muscle cells regardless of the PTH^16^. Thus, acute extracellular changes in ionized calcium may alter cellular function by changing the gradient across the cell membrane, which in turn can alter intracellular calcium. For example, in cardiomyocytes and vascular smooth muscle cells, contraction occurs through the increase in cytoplasmic calcium that comes from the extracellular calcium and/or release from the sarcoplasmic reticulum and mitochondria. Conversely, cytosol calcium efflux leads to relaxation by energy-dependent with adenosine triphosphate (ATP) generation. Thus, acute changes in extracellular ionized calcium may alter the gradient across the cell membrane resulting in unwanted intracellular changes in calcium and cell dysfunction.

### The role of the skeleton in calcium mass transfer

Several studies have shown that a lower d[Ca] acutely increases serum PTH^9^^,^ 10 ^,^ 17 resulting in increased bone turnover. It was previously thought that the variation in serum calcium concentration during dialysis could be a good predictor of calcium mass transfer from the dialysis procedure. Based on this simplistic equation, authors^18^^,^ 19 had determined that d[Ca] of 1.25, 1.5, and 1.75 mmol/L would lead to a negative, neutral and positive calcium balance, respectively, with variable effects on bone turnover. However, calcium balance during hemodialysis is actually hard to predict due to the variables described above, and can be either positive or negative with the same d[Ca] in a given patient. Nevertheless, in general, positive balance is likely when using high (1.75 mmol/L) d[Ca] compared to a low 1.25 mmol/L d[Ca].

One of the confounding factors for the poor accuracy of serum calcium to predict calcium balance is the skeleton. Bone is a reservoir of an exchangeable 300 mg/day of calcium^20^. Almost 20 years ago, Talmage et al.^21^ hypothesized that PTH not only stimulates osteoclast-mediated bone resorption, but also increases the content of some bone surface calcium binding proteins, such as osteocalcin, that could act as calcium buffers. In other words, PTH would increase the amount of osteocalcin in the bone surface, increasing the capacity of the skeleton to acutely donate or retain calcium during dialysis, depending on acute changes in blood calcium. More than 20 years ago, Kurz et al.^22^, using double radiolabeled calcium, showed that the acute bone calcium uptake was higher in high turnover bone disease compared to either mixed uremic osteodystrophy or low turnover bone on a non-dialysis day. Therefore, the skeleton determines not only the exchangeable calcium pool during dialysis but also the pre-dialysis serum calcium through its response to PTH. Indeed, recent studies have proven that calcium balance also varies according to bone turnover status^9^^,^ 10. We and others have shown that calcium balance varies from negative 1500 to positive 800 mg using the same d[Ca] of 1.25 mmol/L^9^^,^ 10.

**Figure 2:**
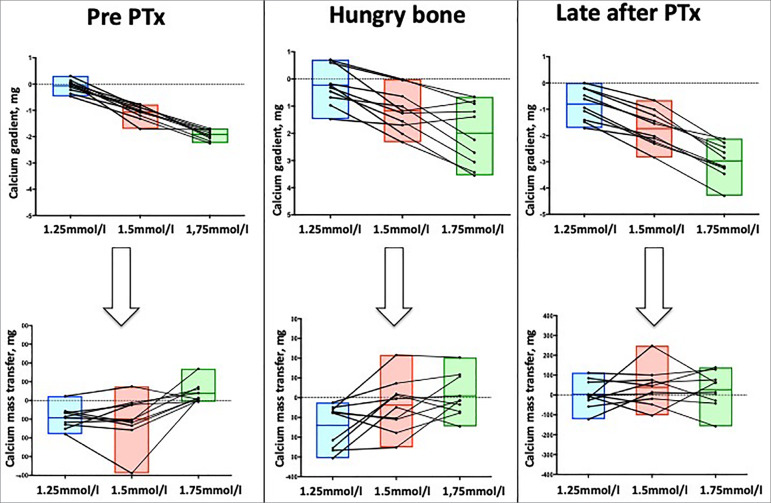
The challenge to predict calcium balance during dialysis based on pre-dialysis serum calcium. Although there is a trend to increase the calcium influx when using higher d[Ca], there is a significant inter-individual variation. Also, there is an intra-individual variation when we compare the same patient in 3 distinct clinical situations: before parathyroidectomy, during hungry bone phase, and later after parathyroidectomy. Calcium gradient and mass transfer using d[Ca] of 1.25mmol/L (blue bars), 1.5mmol/L (red bars), and 1.75 mmol/L (green bars). Data obtained from Goldenstein et al.^9^

Our group has demonstrated the association between bone remodeling markers and calcium mass transfer during a conventional hemodialysis^10^. We studied 23 patients dialyzed using a d[Ca] of 1.0, 1.25, 1.5, and 1.75 mmol/L, in which the mean ± SD and range of calcium removal was -578, -468, 46, and 405 mg, respectively. Multivariate analysis showed that calcium balance was dependent on calcium gradient, PTH, and osteocalcin. Bone remodeling, however, is hard to predict when based only on biomarkers. The ideal study design to evaluate the influence of bone remodeling state on calcium balance would be a repeated analysis in the same patient with different bone turnovers, and maintaining other biases unchanged, i.e., ultrafiltration volume and d[Ca]. We conducted such a study, examining calcium mass transfer in the same patients before parathyroidectomy (PTX), during the first month after surgery in the hungry bone phase, and after surgery when blood calcium stabilized^9^. We confirmed a wide variation of calcium mass transfer during hemodialysis according to each phase (before PTX, hungry bone, and late after PTX) and each d[Ca] used (1.25 vs. 1.5 vs. 1.75 mmol/L). Even with no difference in the ultrafiltration volume, calcium mass transfer varied among phases and among d[Ca] used, supporting our hypothesis of the importance of bone turnover. (Figure 2)

Therefore, the skeleton is a key determinant in net calcium gain or loss during dialysis, and is an important consideration when prescribing the optimal dialysate calcium.

### Effects of calcium balance on myocardium

Cardiovascular (CV) morbidity and mortality rates in patients with ESKD on hemodialysis are alarming with a 5-year survival rate of only 45%. The leading cause of death is cardiovascular events^23^. Alterations in the d[Ca] may be a plausible mechanism to stabilize blood pressure/hemodynamics, myocardial function, hemodynamics, and reduce arrhythmias, especially during and immediately after the dialysis treatment.

The effect of d[Ca] on myocardial perfusion has been studied by a number of methods. Myocardial stunning is common during dialysis, and it can be minimized by the use of a cool dialysate^24^ and the reduction of the ultrafiltration rate, which can be achieved by changing to short daily or long nocturnal hemodialysis sessions^25^. Diastolic dysfunction, an independent predictor of mortality in patients on dialysis, involves abnormal left ventricular relaxation, filling, and distensibility. As coronary blood flow is greater during diastole, diastolic dysfunction may lead to the reduction of coronary perfusion, which may lead to subendocardial ischemia and systolic dysfunction.

Using echocardiography, one study measured diastolic function in hemodialysis with no ultrafiltration on a 1.75 mmol/L d[Ca], finding no impairment in diastolic function despite an increase in ionized calcium^26^. However, two other studies using calcium gluconate infusion^27^ or a high d[Ca] of 1.75 mmol/L^28^ both found impaired diastolic relaxation. Therefore, studies suggest that higher calcium dialysate may worsen ventricular relaxation assessed by echocardiography.

**Figure 3:**
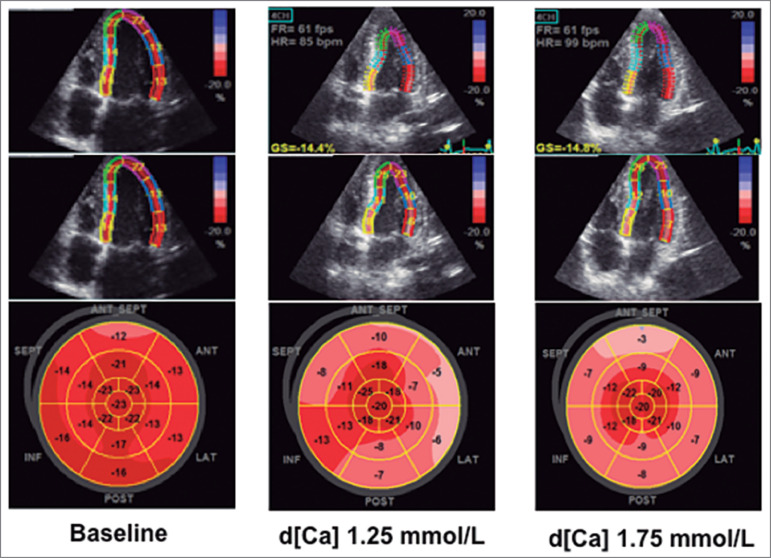
Bull's Eye diagram of four-chamber view and peak longitudinal strain values of all left ventricular segments. The diagram represents the analysis of four-chamber, two-chamber, and left ventricular long axis view, before dialysis (baseline) and at the peak of dialysis (last hour) using d[Ca] of 1.25 mmol/L and 1.75 mmol/L in the same patient, one-week apart. The color-coded map denotes values of peak systolic strain of each segment, with lighter color meaning worse left systolic ventricular dysfunction, which was evident with dialysis, and worse using d[Ca] 1.75 mmol/L.

Recently, our group used a more sensitive method, two dimensional speckle imaging with strain analysis, to evaluate the impact of d[Ca] on myocardial performance during hemodialysis^29^. We found hypercalcemia (11.5 ± 0.8 mg/dL) after hemodialysis using d[Ca] of 1.75 mmol/L and improved hemodynamic stability in terms of blood pressure. However, the global longitudinal strain (GLS) was worse during the last hour of hemodialysis compared to baseline (p < 0.001). In addition, the GLS was worse with d[Ca] of 1.75 than 1.25 mmol/L (−16.1 ± 2.6% *vs.* −17.3 ± 2.9%, respectively; *p* < 0.001). Multiple linear regression showed that independent risk factors for GLS were transferrin, c-reactive protein, baseline GLS, weight loss during hemodialysis, and post dialysis serum calcium. Figure 3 shows echocardiogram images illustrating GLS at baseline and at the peak hemodialysis using d[Ca] 1.25 mmol/L and d[Ca] 1.75 mmol/L (bull's eye graphical representation).

Previous studies have shown an increased calcium-induced myocardial contractility when a d[Ca] of 1.75 mmol/L was employed^30^^,^ 31. However, one of these studies was performed in 1984^30^ and included only eight patients using three different d[Ca] to evaluate the left ventricular contractility by two-dimensional echocardiography. Authors concluded that the increase of ionized calcium after dialysis was associated with an improvement of contractility. Other authors four years later^31^ included seven patients and tested 3 different d[Ca]. Left ventricular contractility was assessed using the relation between left ventricular end-systolic wall stress and myocardial systolic performance. They concluded that high d[Ca] had a positive impact of myocardial performance. A recent study has evaluated cardiac function in a Langendorff-like system of a zebrafish^32^. By manipulating the calcium concentration of the perfusion buffer, authors surprisingly found that the ejection fraction initially increased along with the increase in calcium concentration, similarly to previously mentioned studies, and then decreased. Although the experimental scenario is unlike clinical practice, this finding should raise alert about the deleterious effect of high d[Ca]. There is no doubt that calcium is imperative to ventricular contraction. These inconsistent findings suggest that calcium is indeed important for effective myocardial contraction in an almost direct relationship, although hypercalcemia can impair ventricular function.

The most common cause of cardiovascular death in patients undergoing dialysis is arrhythmias. In an analysis of a large dialysis provider dataset, low calcium dialysate (< 1.25 mmol/L), and increased dialysate calcium gradient were associated with a 2 and 1.4 odds ratio, respectively, of sudden cardiac arrest^33^. Arrhythmias may be more common with low versus high d[Ca], but increased sympathetic activity that may trigger arrhythmias may be more common with high, compared to low, d[Ca]. The effect of long term d[Ca] concentration on cardiovascular outcomes is not well studied. However, a recent secondary analyses of the EVOLVE trial found that baseline dialysate calcium concentration, which was generally higher in Europe and Latin America, did not alter the outcomes of the trial that compared cinacalcet to placebo on cardiovascular composite outcome^34^.

### Effects of calcium balance on vascular calcification

Arterial calcification is common as over 70% of patients starting dialysis have significant coronary artery calcification on CT scanning^35^^,^ 36. The pathology of arterial calcification includes a mix of atherosclerotic calcification and medial calcification. In dialysis patients who died of a cardiovascular event, atherosclerotic plaque was more calcified than age-matched controls with similar cause of death^37^. Similarly, the prevalence of medial calcification of large arteries increases with progression of kidney disease and is higher in patients on dialysis than age matched controls^38^. Aorta calcification can lead to increased pulse pressure and increased cardiac afterload resulting in increased cardiovascular events and mortality^39^.

The mechanism of medial calcification is a multi-step process initiated by dedifferentiation of vascular smooth muscle cells (VSMC) to become osteo-chondrocytic-like cells via upregulation of the transcription factor RUNX2. These transformed cells then mineralize in a manner similar to bone, with production of collagen and non-collagenous proteins on which secreted matrix vesicles (containing multiple proteins and calcium and phosphate) mineralize. Vascular calcification is regulated in part by inhibitors of calcification including fetuin-A, a circulating reverse acute phase reactant protein that acts to bind circulating 'calcioproteins' containing calcium and phosphate for clearance, and matrix-gla protein, an inhibitor that is upregulated in vascular smooth muscle cells^40^.

Positive calcium balance and/or hypercalcemia are involved in the pathogenesis of arterial calcification. VSMC move from a contractile to synthetic phenotype, a prerequisite for de-differentiation. In rats with CKD, freshly isolated VSMC from aorta have increased intracellular calcium concentration, indicative of the synthetic state^16^. These cells then upregulate RUNX2 to become osteo-chondrocytic-like cells in the presence of uremic serum^41^. Shanahan and colleagues demonstrated that calcium induces release of matrix vesicles and increased calcification independently and synergistically with phosphate in cultured VSMC^42^. Giachelli's group also demonstrated that calcium-induced calcification was synergistic with hyperphosphatemia. Furthermore, they demonstrated that incubating VSMC with high calcium media led to upregulation of Pit-1, a sodium-phosphate transporter important in the upregulation of RUNX^43^. *In vivo*, calcium containing phosphate binders induce arterial calcification in 5/6^th^ nephrectomy rats^44^, Cy/+ model of CKD^45^, and the LDLR-/- high-fat-fed mice with CKD^46^ despite lower levels of serum phosphate. In our Cy/+ rat model of progressive kidney disease, we treated rats with advanced CKD with calcium administered in drinking water, calcimimetic R-568, and R-568 plus calcium versus no treatment^47^. Treatment with calcium in the drinking water led to increased thoracic aorta, heart, and aortic valve calcification regardless of the serum level of calcium, indicating positive calcium exposure/balance can induce arterial calcification regardless of calcium blood levels. The calcium treatment led to an even greater calcification than observed with hyperphosphatemia and normal calcium levels^47^. Thus, hypercalcemia, or positive calcium balance even without hypercalcemia, can directly induce calcification *in vitro* and *in vivo*.

In patients receiving hemodialysis, most randomized trials comparing calcium-based phosphate binders, compared to non-calcium binders, show greater progression of coronary artery calcification^48^. However, studies examining the role of calcium load from dialysate calcium are limited. A small study compared dialyzed patients against three acute variations of calcium concentrations and found increased carotid-femoral and carotid-radial pulse wave velocity (PWV; a measure of increased stiffness) with higher dialysate calcium^49^. Another small study randomized patients on nocturnal dialysis to low calcium dialysate (1.3 mmol/L, n = 24) or high calcium dialysate (1.6 or 1.75 mmol/L, n = 26) and found no difference in abdominal aorta calcification by CT over one year^50^. However, Ok and colleagues (17) conducted a large randomized trial in patients on thrice weekly HD with intact parathyroid hormone levels ≤ 300 randomized to 1.25-mmol/L Ca dialysate (n = 212) or 1.75-mmol/L Ca dialysate (n = 213). The results showed a significant increase in coronary artery calcification in the patients randomized to 1.75 mmol/L Ca dialysate compared to the lower dialysate calcium. Importantly, hyperphosphatemia also increased coronary artery calcification, and the combination of hyperphosphatemia and high calcium dialysate was additive in inducing increased coronary artery calcification. Thus, increased calcium dialysate, especially in the setting of hyperphosphatemia, appears to increase arterial calcification in hemodialysis patients, similar to observations from *in vitro* VSMC cultures and *in vivo* in rodent models of CKD.

## Conclusion

The d[Ca] can cause acute hypercalcemia in a given patient, depending on ultrafiltration volume and bone turnover status. However, the role of d[Ca] causing a positive calcium balance as a primary cause of systemic organ damage is not well appreciated. This review addressed potential associations between d[Ca] and systemic changes in the skeleton, myocardial, and vessels. The best d[Ca] remains unknown as both low and high d[Ca] can improve or deteriorate organ function. As an example, while low d[Ca] can be a good choice for patients with adynamic bone disease to avoid vascular calcification, it can also induce hypocalcemia and stimulate PTH secretion, leading to high risk of arrhythmias and cardiac arrest. On the other hand, high d[Ca] can lead to better hemodynamic stability, although this may increase the risk of calcification, suppress the PTH secretion, and cause ventricular dysfunction during hemodialysis^9^^,^ 10 ^,^ 29 ^,^ 51

It is difficult to distinguish between the direct effect of d[Ca] versus indirect effect of d[Ca] on hypercalcemia. However, since d[Ca] is a major determinant of serum calcium levels, or at least acute changes during dialysis, there is a need for more research to fully elucidate the impact of d[Ca] on systemic organ damage and to establish a direct cause-effect relationship. However, the accumulated data to date does support that central pumping of specific d[Ca] in outpatient dialysis units is not a good practice, as one size does not fit all.

## References

[1] Chesnaye NC, Schaefer F, Bonthuis M, Holman R, Baiko S, Baskin E (2017). Mortality risk disparities in children receiving chronic renal replacement therapy for the treatment of end-stage renal disease across Europe: an ESPN-ERA/EDTA registry analysis. Lancet.

[2] Vautour L, Goltzman D, Bilezikian JP (2018). Primer on the metabolic bone diseases and disorders of mineral metabolism.

[3] Isakova T, Xie H, Yang W, Xie D, Anderson AH, Scialla J (2011). Fibroblast growth factor 23 and risks of mortality and end-stage renal disease in patients with chronic kidney disease. JAMA.

[4] Hill KM, Martin BR, Wastney ME, McCabe GP, Moe SM, Weaver CM (2013). Oral calcium carbonate affects calcium but not phosphorus balance in stage 3-4 chronic kidney disease. Kidney Int.

[5] Spiegel DM, Brady K (2012). Calcium balance in normal individuals and in patients with chronic kidney disease on low- and high-calcium diets. Kidney Int.

[6] Ramalho JPEM, Takeichi APM, Moyses RMA, Titan SM (2019). Calcitriol and FGF-23, but not PTH nor sclerostin, are associated with calciuria in CKD. Int Urol Nephrol.

[7] Nordin BE (1990). Calcium homeostasis. Clin Biochem.

[8] Goransson LG, Skadberg O, Bergrem H (2005). Albumin-corrected or ionized calcium in renal failure? What to measure?. Nephrol Dial Transplant.

[9] Goldenstein PT, Graciolli FG, Antunes GL, Dominguez WV, Reis LM, Moe S (2018). A prospective study of the influence of the skeleton on calcium mass transfer during hemodialysis. PLoS One.

[10] Karohl C, Paschoal JP, Castro MC, Elias RM, Abensur H, Romão JE (2010). Effects of bone remodelling on calcium mass transfer during haemodialysis. Nephrol Dial Transplant.

[11] Bacchetta J, Sellier-Leclerc AL, Bertholet-Thomas A, Carlier MC, Cartier R, Cochat P (2015). Calcium balance in pediatric online hemodiafiltration: beware of sodium and bicarbonate in the dialysate. Nephrol Ther.

[12] Argiles A, Kerr PG, Canaud B, Flavier JL, Mion C (1993). Calcium kinetics and the long-term effects of lowering dialysate calcium concentration. Kidney Int.

[13] Basile C, Giordano R, Montanaro A, Maio PD, Padova FD, Marangi AL (2001). Effect of acetate-free biofiltration on the anaemia of haemodialysis patients a prospective cross-over study. Nephrol Dial Transplant.

[14] Grundstrom G, Christensson A, Alquist M, Nilsson LG, Segelmark M (2013). Replacement of acetate with citrate in dialysis fluid: a randomized clinical trial of short term safety and fluid biocompatibility. BMC Nephrol.

[15] Zhang YB, Smogorzewski M, Ni Z, Massry SG (1994). Altered cytosolic calcium homeostasis in rat cardiac myocytes in CRF. Kidney Int.

[16] Rodenbeck SD, Zarse CA, McKenney-Drake ML, Bruning RS, Sturek M, Chen NX (2017). Intracellular calcium increases in vascular smooth muscle cells with progression of chronic kidney disease in a rat model. Nephrol Dial Transplant.

[17] Ok E, Asci G, Bayraktaroglu S, Toz H, Ozkahya M, Yilmaz M (2016). Reduction of dialysate calcium level reduces progression of coronary artery calcification and improves low bone turnover in patients on hemodialysis. J Am Soc Nephrol.

[18] Di Filippo S, Carfagna F, La Milia V, Bellasi A, Casagrande G, Bianchi C (2018). Assessment of intradialysis calcium mass balance by a single pool variable-volume calcium kinetic model. Hemodial Int.

[19] Gotch FA, Kotanko P, Thijssen S, Levin NW (2010). The KDIGO guideline for dialysate calcium will result in an increased incidence of calcium accumulation in hemodialysis patients. Kidney Int.

[20] Messa P (2013). The ups and downs of dialysate calcium concentration in haemodialysis patients. Nephrol Dial Transplant.

[21] Talmage RV, Matthews JL, Mobley HT, Lester GE (2003). Calcium homeostasis and bone surface proteins, a postulated vital process for plasma calcium control. J Musculoskelet Neuronal Interact.

[22] Kurz P, Monier-Faugere MC, Bognar B, Werner E, Roth P, Vlachojannis J (1994). Evidence for abnormal calcium homeostasis in patients with adynamic bone disease. Kidney Int.

[23] Wheeler DC, London GM, Parfrey PS, Block GA, Correa-Rotter R, Dehmel B (2014). Effects of cinacalcet on atherosclerotic and nonatherosclerotic cardiovascular events in patients receiving hemodialysis: the EValuation Of Cinacalcet HCl Therapy to Lower CardioVascular Events (EVOLVE) trial. J Am Heart Assoc.

[24] Jefferies HJ, Burton JO, McIntyre CW (2011). Individualised dialysate temperature improves intradialytic haemodynamics and abrogates haemodialysis-induced myocardial stunning, without compromising tolerability. Blood Purif.

[25] Jefferies HJ, Virk B, Schiller B, Moran J, McIntyre CW (2011). Frequent hemodialysis schedules are associated with reduced levels of dialysis-induced cardiac injury (myocardial stunning). Clin J Am Soc Nephrol.

[26] Ie EH, Vletter WB, Cate FJ, Weimar W, Zietse R (2004). Increase in serum ionized calcium during diffusive dialysis does not affect left ventricular diastolic function. Blood Purif.

[27] Virtanen VK, Saha HH, Groundstroem KW, Seppala ES, Pasternack AI (1998). Calcium infusion and left ventricular diastolic function in patients with chronic renal failure. Nephrol Dial Transplant.

[28] Nappi SE, Saha HH, Virtanen VK, Mustonen JT, Pasternack AI (1999). Hemodialysis with high-calcium dialysate impairs cardiac relaxation. Kidney Int.

[29] Silva VB, Macedo TA, Braga TMS, Silva BC, Graciolli FG, Dominguez WV (2019). High dialysate calcium concentration is associated with worsening left ventricular function. Sci Rep.

[30] Henrich WL, Hunt JM, Nixon JV (1984). Increased ionized calcium and left ventricular contractility during hemodialysis. N Engl J Med.

[31] Lang RM, Fellner SK, Neumann A, Bushinsky DA, Borow KM (1988). Left ventricular contractility varies directly with blood ionized calcium. Ann Intern Med.

[32] Zhang H, Dvornikov AV, Huttner IG, Ma X, Santiago CF, Fatkin D (2018). A Langendorff-like system to quantify cardiac pump function in adult zebrafish. Dis Model Mech.

[33] Pun PH, Horton JR, Middleton JP (2013). Dialysate calcium concentration and the risk of sudden cardiac arrest in hemodialysis patients. Clin J Am Soc Nephrol.

[34] Pun PH, Abdalla S, Block GA, Chertow GM, Correa-Rotter R, Dehmel B (2016). Cinacalcet, dialysate calcium concentration, and cardiovascular events in the EVOLVE trial. Hemodial Int.

[35] Tomiyama C, Higa A, Dalboni MA, Cendoroglo M, Draibe SA, Cuppari L (2006). The impact of traditional and non-traditional risk factors on coronary calcification in pre-dialysis patients. Nephrol Dial Transplant.

[36] Barreto DV, Barreto FC, Carvalho AB, Cuppari L, Draibe SA, Dalboni MA (2008). Association of changes in bone remodeling and coronary calcification in hemodialysis patient:s a prospective study. Am J Kidney Dis.

[37] Schwarz U, Buzello M, Ritz E, Stein G, Raabe G, Wiest G (2000). Morphology of coronary atherosclerotic lesions in patients with end-stage renal failure. Nephrol Dial Transplant.

[38] Biyik Z, Selcuk NY, Tonbul HZ, Anil M, Uyar M (2016). Assessment of abdominal aortic calcification at different stages of chronic kidney disease. Int Urol Nephrol.

[39] Niu Q, Hong Y, Lee CH, Men C, Zhao H, Zuo L (2018). Abdominal aortic calcification can predict all-cause mortality and CV events in dialysis patients: a systematic review and meta-analysis. PLoS One.

[40] Lomashvili KA, Wang X, Wallin R, O'Neill WC (2011). Matrix Gla protein metabolism in vascular smooth muscle and role in uremic vascular calcification. J Biol Chem.

[41] Moe SM, Duan D, Doehle BP, O'Neill KD, Chen NX (2003). Uremia induces the osteoblast differentiation factor Cbfa1 in human blood vessels. Kidney Int.

[42] Reynolds JL, Joannides AJ, Skepper JN, McNair R, Schurgers LJ, Proudfoot D (2004). Human vascular smooth muscle cells undergo vesicle-mediated calcification in response to changes in extracellular calcium and phosphate concentrations: a potential mechanism for accelerated vascular calcification in ESRD. J Am Soc Nephrol.

[43] Yang H, Curinga G, Giachelli CM (2004). Elevated extracellular calcium levels induce smooth muscle cell matrix mineralization in vitro. Kidney Int.

[44] Cozzolino M, Staniforth ME, Liapis H, Finch J, Burke S, Dusso A (2003). Sevelamer hydrochloride attenuates kidney and cardiovascular calcifications in long-term experimental uremia. Kidney Int.

[45] Moe SM, Chen NX, Newman CL, Gattone VH, Organ JM, Chen X (2014). A comparison of calcium to zoledronic acid for improvement of cortical bone in an animal model of CKD. J Bone Miner Res.

[46] Davies MR, Lund RJ, Mathew S, Hruska KA (2005). Low turnover osteodystrophy and vascular calcification are amenable to skeletal anabolism in an animal model of chronic kidney disease and the metabolic syndrome. J Am Soc Nephrol.

[47] Moe SM, Seifert MF, Chen NX, Sinders RM, Chen X, Duan D (2009). R-568 reduces ectopic calcification in a rat model of chronic kidney disease-mineral bone disorder (CKD-MBD). Nephrol Dial Transplant.

[48] Block GA, Spiegel DM, Ehrlich J, Mehta R, Lindbergh J, Dreisbach A (2005). Effects of sevelamer and calcium on coronary artery calcification in patients new to hemodialysis. Kidney Int.

[49] LeBeouf A, Mac-Way F, Utescu MS, Chbinou N, Douville P, Desmeules S (2009). Effects of acute variation of dialysate calcium concentrations on arterial stiffness and aortic pressure waveform. Nephrol Dial Transplant.

[50] Masterson R, Blair S, Polkinghorne KR, Lau KK, Lian M, Strauss BJ (2017). Low versus high dialysate calcium concentration in alternate night nocturnal hemodialysis: a randomized controlled trial. Hemodial Int.

[51] Jimenez ZN, Silva BC, Reis LD, Castro MC, Ramos CD, Costa-Hong V (2016). High dialysate calcium concentration may cause more sympathetic stimulus during hemodialysis. Kidney Blood Press Res.

[52] Ogden DA, Holmes JH (1966). Changes in total and ultrafilterable plasma calcium and magnesium during hemodialysis. Trans Am Soc Artif Intern Organs.

[53] Wing AJ (1968). Optimum calcium concentration of dialysis fluid for maintenance haemodialysis. Br Med J.

[54] Goldsmith RS, Furszyfer J, Johnson WJ, Fournier AE, Arnaud CD (1971). Control of secondary hyperparathyroidism during long-term hemodialysis. Am J Med.

[55] Strong HE, Schatz BC, Shinaberger JH, Coburn JW (1971). Measurement of dialysance and bi-directional fluxes of calcium in vivo using radiocalcium. Trans Am Soc Artif Intern Organs.

[56] Skrabal F, Dittrich P, Gabl F (1974). Calcium uptake and phosphate removal during hemodialysis with variing dialysate calcium. Klin Wschr.

[57] Carney SL, Gillies AH (1985). Effect of an optimum dialysis fluid calcium concentration on calcium mass transfer during maintenance hemodialysis. Clin Nephrol.

[58] Hou SH, Zhao J, Ellman CF, Hu J, Griffin Z, Spiegel DM (1991). Calcium and phosphorus fluxes during hemodialysis with low calcium dialysate. Am J Kidney Dis.

[59] Malberti F, Corradi B, Tetta C, Imbasciati E (1994). Calcium balance and serum ionized calcium fluctuations in on-line haemodiafiltration in relation to ultrafiltration rate and dialysate calcium concentration. Nephrol Dial Transplant.

[60] Argiles A, Mion CM (1995). Calcium balance and intact PTH variations during haemodiafiltration. Nephrol Dial Transplant.

[61] Fabrizi F, Bacchini G, Di Filippo S, Pontoriero G, Locatelli F (1996). Intradialytic calcium balances with different calcium dialysate levels Effects on cardiovascular stability and parathyroid function. Nephron.

[62] Ding F, Ahrenholz P, Winkler RE, Ramlow W, Tiess M, Michelsen A (2002). Online hemodiafiltration versus acetate-free biofiltration: a prospective crossover study. Artif Organs.

[63] Al-Hejaili F, Kortas C, Leitch R, Heidenheim AP, Clement L, Nesrallah G (2003). Nocturnal but not short hours quotidian hemodialysis requires an elevated dialysate calcium concentration. J Am Soc Nephrol.

[64] Sigrist M, McIntyre CW (2006). Calcium exposure and removal in chronic hemodialysis patients. J Ren Nutr.

[65] Basile C, Libutti P, Di Turo AL, Tundo S, Maselli P, Casucci F (2011). Calcium mass balances during standard bicarbonate hemodialysis and long-hour slow-flow bicarbonate hemodialysis. J Nephrol.

[66] Bosticardo G, Malberti F, Basile C, Leardini L, Libutti P, Filiberti O (2012). Optimizing the dialysate calcium concentration in bicarbonate haemodialysis. Nephrol Dial Transplant.

[67] Basile C, Libutti P, Di Turo AL, Vernaglione L, Casucci F, Losurdo N (2012). Effect of dialysate calcium concentrations on parathyroid hormone and calcium balance during a single dialysis session using bicarbonate hemodialysis: a crossover clinical trial. Am J Kidney Dis.

[68] Safranek R, Moucka P, Vavrova J, Palicka V, Pavlikova L, Sulkova SD (2015). Changes of serum calcium, magnesium and parathyroid hormone induced by hemodialysis with citrate-enriched dialysis solution. Kidney Blood Press Res.

[69] Tiranathanagul K, Tangvoraphonkchai K, Srisawat N, Susantitaphong P, Tungsanga K, Praditpornsilpa K (2015). Acute intradialytic cardiac function and inflammatory cytokine changes during high-efficiency online hemodiafiltration with acetate-free and standard dialysis solutions. Ther Apher Dial.

[70] Waniewski J, Debowska M, Wojcik-Zaluska A, Ksiazek A, Zaluska W (2016). Quantification of dialytic removal and extracellular calcium mass balance during a weekly cycle of hemodialysis. PLoS One.

[71] Havlin J, Vankova S (2019). Intradialytic alkalinization is a neglected factor affecting calcium mass balance and parathyroid hormone level during haemodiafiltration. Clin Kidney J.

